# Clinical and Genetic Findings in *CTNNA1*-Associated Macular Pattern Dystrophy

**DOI:** 10.1016/j.ophtha.2020.10.032

**Published:** 2021-06

**Authors:** Alexander Tanner, Hwei Wuen Chan, Jose S. Pulido, Gavin Arno, Rola Ba-Abbad, Neringa Jurkute, Anthony G. Robson, Catherine A. Egan, Hannah Knight, Antonio Calcagni, Rachel L. Taylor, Eva Lenassi, Graeme C. Black, Anthony T. Moore, Michel Michaelides, Andrew R. Webster, Omar A. Mahroo

**Affiliations:** 1 UCL Institute of Ophthalmology, University College London, London, United Kingdom; 2 Moorfields Eye Hospital, London, United Kingdom; 3 Department of Ophthalmology, National University Hospital, Singapore, Republic of Singapore; 4 Manchester Centre for Genomic Medicine, St. Mary’s Hospital, Manchester, United Kingdom; 5 Division of Evolution and Genomic Sciences, School of Biological Sciences, Faculty of Biology, Medicines and Health, University of Manchester, Manchester, United Kingdom; 6 Department of Ophthalmology, University of California, San Francisco, School of Medicine, San Francisco, California; 7 Section of Ophthalmology, King’s College London, St. Thomas’ Hospital, London, United Kingdom; 8 Physiology, Development and Neuroscience, University of Cambridge, Cambridge, United Kingdom; 9 Retina Service and Vickie and Jack Farber Vision Research Center, Wills Eye Hospital, Philadelphia, Pennsylvania

**Keywords:** Electrooculography, Electroretinography, Macular degeneration, Macular pattern dystrophy, Retina, Retinal dystrophies

Macular pattern dystrophies of the retinal pigment epithelium have various causes and effects on vision, with abnormalities particularly evident on short-wavelength autofluorescence imaging. A number of genes have been implicated, frequently *PRPH2*. In 2015, 3 heterozygous missense variants in *CTNNA1* (encoding the widely expressed α-catenin protein) were associated with butterfly-shaped pigment dystrophy: c.953T→C (p.Leu318Ser), c.1293T→G (p.Ile431Met), and c.919G→A (p.Glu307Lys).^1^ Herein, we describe 6 additional families with *CTNNA1* missense variants (affected individuals exhibiting pattern dystrophy), corroborating the previous report. Four previously unreported variants are described, with predicted effects on protein structure. We also show near-infrared reflectance findings.

Electronic patient records from Moorfields Eye Hospital, London, United Kingdom, and Manchester Centre for Genomic Medicine, Manchester, United Kingdom, were inspected to identify individuals with inherited retinopathy associated with variants in *CTNNA1*. The study had review board approval (Moorfields Eye Hospital R&D department and North London and Greater Manchester West Research Ethics Committees) and conformed to the tenets of the Declaration of Helsinki. The requirement for informed consent was waived because of the retrospective nature of the study.

**Figure 1:**
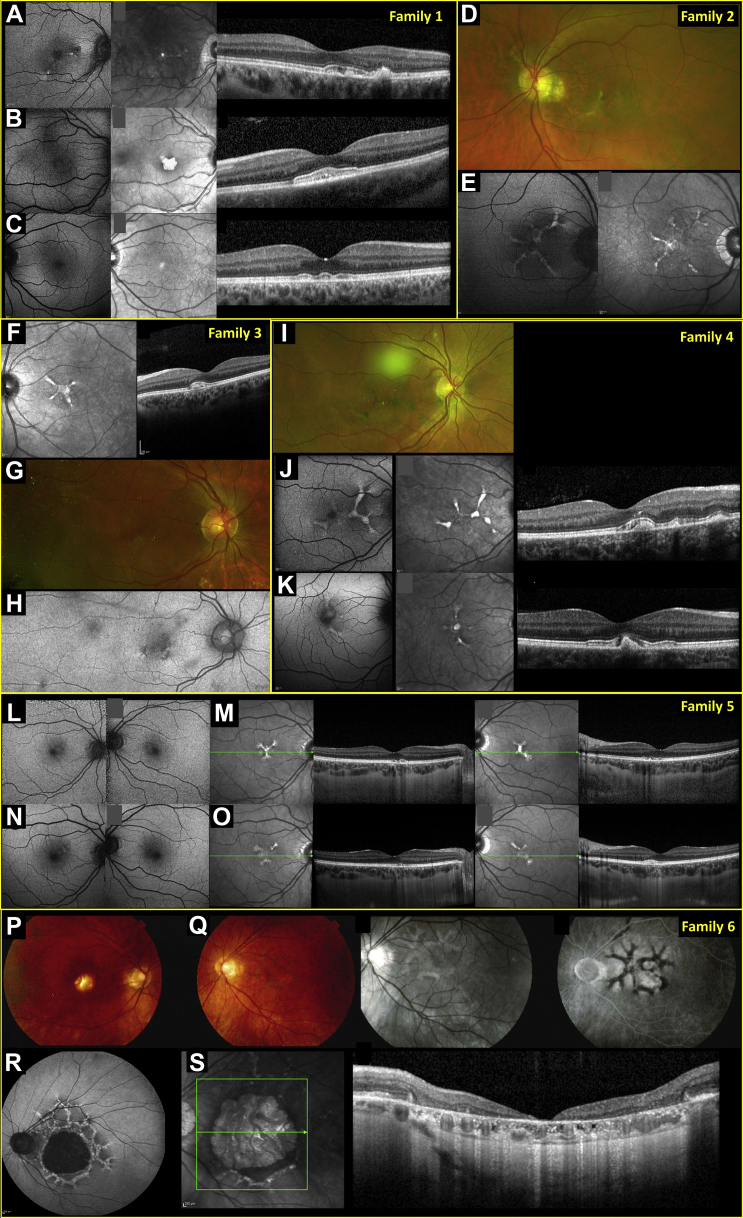
Multimodal imaging findings: (**A**–**C**) family 1, (**D**, **E**) family 2, (**F**–**H**) family 3, (**I**–**K**) family 4, (**L**–**O**) family 5, and (**P**–**S**) family 6. **A**–**C**, Images from family 1 (**A**) proband, (**B**) her younger daughter, 4 years of age, and (**C**) older daughter, 19 years of age. Panels show short-wavelength autofluorescence (SW-AF; leftmost panels: right eye of proband and younger daughter, left eye of older daughter), near infrared reflectance (NIR; middle panels: same eyes as for SW-AF), and OCT (right panels: right eye of proband, left eye of younger daughter, right eye of older daughter). **D**, **E**, Images from proband of family 2: (**D**) pseudocolor image (left eye) showing hyperpigmented lines radiating from fovea and (**E**) SW-AF (left panel) and NIR (right panel) images from the right macula. Both eyes show peripapillary atrophy. **F**–**H**, Images from family 3: (**F**) left eye of proband (NIR and OCT) and (**G**, **H**) right eye of her mother (**G**, pseudocolor; **H**, green autofluorescence). **I**–**K**, Images from family 4: (**I**) pseudocolor; (**J**) SW-AF (left panel), NIR (middle panel), and OCT (right panel) images from the right eye of the proband; (**K**) SW-AF, NIR, and OCT images from the right macula of the proband’s male affected cousin. Abnormalities appear more evident on NIR compared with SW-AF images. **L**–**O**, Images from the proband from family 5: (**L**) SW-AF images and (**M**) NIR and OCT images from both eyes of the patient, 31 years of age; (**N**, **O**) Corresponding images obtained 3 years later. **P**–**S**, Images from the proband from family 6 at 62 years of age: (**P**) color fundus image of right eye; (**Q**) color, red-free, and fundus fluorescein images from the left eye; and at 78 years of age: (**R**) SW-AF image of the left eye and (**S**) NIR reflectance and OCT images.

We identified 11 affected patients (8 female) from 6 unrelated families (nonconsanguineous pedigrees; Fig S1, available at www.aaojournal.org). Demographics, genotypes, and clinical features, including electrophysiologic features, are summarized in Table S1 (available at www.aaojournal.org). Median age at presentation was 34 years (range, 4–63). Follow-up ranged from 0 to 18 years. Figure 1 depicts multimodal imaging; clinical histories are below.

The proband from family 1 was a 43-year-old woman with mild symptoms, including difficulty driving in low light levels. Visual acuities were 20/20 (right eye) and 20/17 (left eye). Macular pigmentary changes were noted. Five months later, recorded acuity was 20/30 bilaterally. Her 4-year-old daughter was referred because her ophthalmologist had observed macular changes (more marked than in her mother). Visual acuity was 20/40 bilaterally, and she had hyperopic astigmatism. Three months later, recorded visual acuity was 20/30 bilaterally. The proband’s older daughter, 19 years of age, was asymptomatic, with visual acuity of 20/20 bilaterally. Retinal imaging revealed subtle changes (Fig 1C).

The proband from family 2 was a 43-year-old man reporting distortion in right-eye vision. Medical history included asthma, vitamin D deficiency, and thyroid cancer. Visual acuity was 20/17 bilaterally. Hyperpigmented lines radiating from the fovea (Fig 1D) were noted bilaterally. Eighteen years after presentation, visual acuities were 20/60 (right eye) and 20/30 (left eye). His sister, 38 years of age, was referred by her optometrist, who noted bilateral retinal changes. Her visual acuity was 20/20 bilaterally. Subtle macular pigmentary changes and peripheral scalloped atrophic areas were noted. Visual acuities were unchanged 11 months later.

The proband from family 3 was a 27-year-old asymptomatic woman referred by her optometrist after discovery of retinal abnormalities. Visual acuity was 20/20 bilaterally. Fundus examination showed macular pigmentary changes (Fig 1F). Twenty-one months later, acuities and retinal findings were unchanged. Her mother, also asymptomatic, was examined at 63 years of age and showed macular pigmentary disturbance (Fig 1G,H) with peripheral drusen.

The proband from family 4 was a 32-year-old woman whose optometrist found retinal changes. She reported mild difficulties in low light levels. Visual acuities were 20/30 (right eye) and 20/17 (left eye). Linear pigmentary changes were seen centrally in both fundi. Ten months later, visual acuities were unchanged. Her maternal male cousin was reviewed at 34 years of age. Visual acuities were 20/30 (right eye) and 20/20 (left eye). Fundus examination showed similar changes (Fig 1K). Recorded acuities were 20/20 (right eye) and 20/17 (left eye) 19 months later. Other relatives were not evaluated in our service, but affected individuals had been diagnosed elsewhere (maternal uncle and maternal female cousin).

The proband from family 5 was a 31-year-old woman who reported mildly distorted central vision. She showed peripheral retinal pigmentary changes and mentioned a diagnosis of retinitis pigmentosa from a different hospital. She later described possible difficulties in dim light, occasionally bumping into things. Medical history included postural orthostatic tachycardia syndrome and hypermobility. Her maternal grandfather had been reported to have had “tunnel vision” (later losing central vision), and her maternal grandmother had been diagnosed with macular degeneration. Examination revealed visual acuity of 20/20 and macular pigmentary changes (Fig 1L,M) bilaterally, with peripheral reticular changes at the level of the retinal pigment epithelium. Normal electroretinography images made retinitis pigmentosa unlikely. Four years later, visual acuity was 20/17 bilaterally and imaging findings were similar (Fig 1N,O), with short-wavelength autofluorescence abnormalities slightly more evident than at first visit.

The proband from family 6 was a 62-year-old man reporting a 2-year history of right-eye visual loss. Visual acuities were 20/125 (right eye) and 20/30 (left eye). Foveal atrophy was noted in the right fundus, and pigmentary mottling of the left macula was evident (Fig 1Q). Macular pattern dystrophy was diagnosed. At his latest visit at 79 years of age, visual acuities were recorded as 20/80 (right eye) and 20/200 (left eye), with marked macular atrophy bilaterally.

Genetic testing is detailed in Table S1. Families 1 and 3 showed the c.965C→T (p.Ser322Leu) variant in *CTNNA1*; families 2 and 4 showed the c.1316C→T (p.Ser439Phe) variant; and the ninth and tenth patients (families 5 and 6) showed c.1294G→A (p.Glu432Lys) and c.973A→G (p.Thr325Ala) variants, respectively. All were heterozygous, deemed “probably damaging” (PolyPhen-2)^2^ or “disease causing” (MutationTaster),^3^ and were absent in more than 60 000 probands in the gnomAD 2.1 database.^4^ Figure S2 (available at www.aaojournal.org) illustrates protein structure, showing proximity to previously reported variants. No variants were found in other genes that could explain the phenotype (despite whole genome^5^^,^^6^ or exome sequencing, or multiple gene panel testing).

In our cohort, visual symptoms were mild or absent (with pattern electroretinography evidence of preserved macular function) in all but 1 patient. Long-term visual prognosis is unknown. Abnormalities, particularly in younger patients, seemed more evident on near-infrared reflectance than on autofluorescence. Electrophysiologic analysis revealed normal generalized rod and cone system function, but a high incidence of generalized retinal pigment epithelium dysfunction (largely consistent with the previous report^1^).

Our study was retrospective, and unaffected individuals were not evaluated, precluding determination of segregation or penetrance. Skipped generations or lack of positive family history could represent incomplete penetrance or simply absence of symptoms. The family 6 patient differed from the others, with more severely reduced vision and central retinal atrophy. This may relate to his particular *CTNNA1* variant, his age, or other modifiers, including possible coincidental age-related maculopathy. Further cases will help determine genotype–phenotype correlations.
