# Supplementary material for: Clinical and Genetic Findings in CTNNA1-Associated Macular Pattern Dystrophy
Source: Ophthalmology. 2021 Jun;128(6):952–5. doi: 10.1016/j.ophtha.2020.10.032 (PMC8162661; doi:10.1016/j.ophtha.2020.10.032)
Supplement: Figure S1 [file mmc1.pdf]

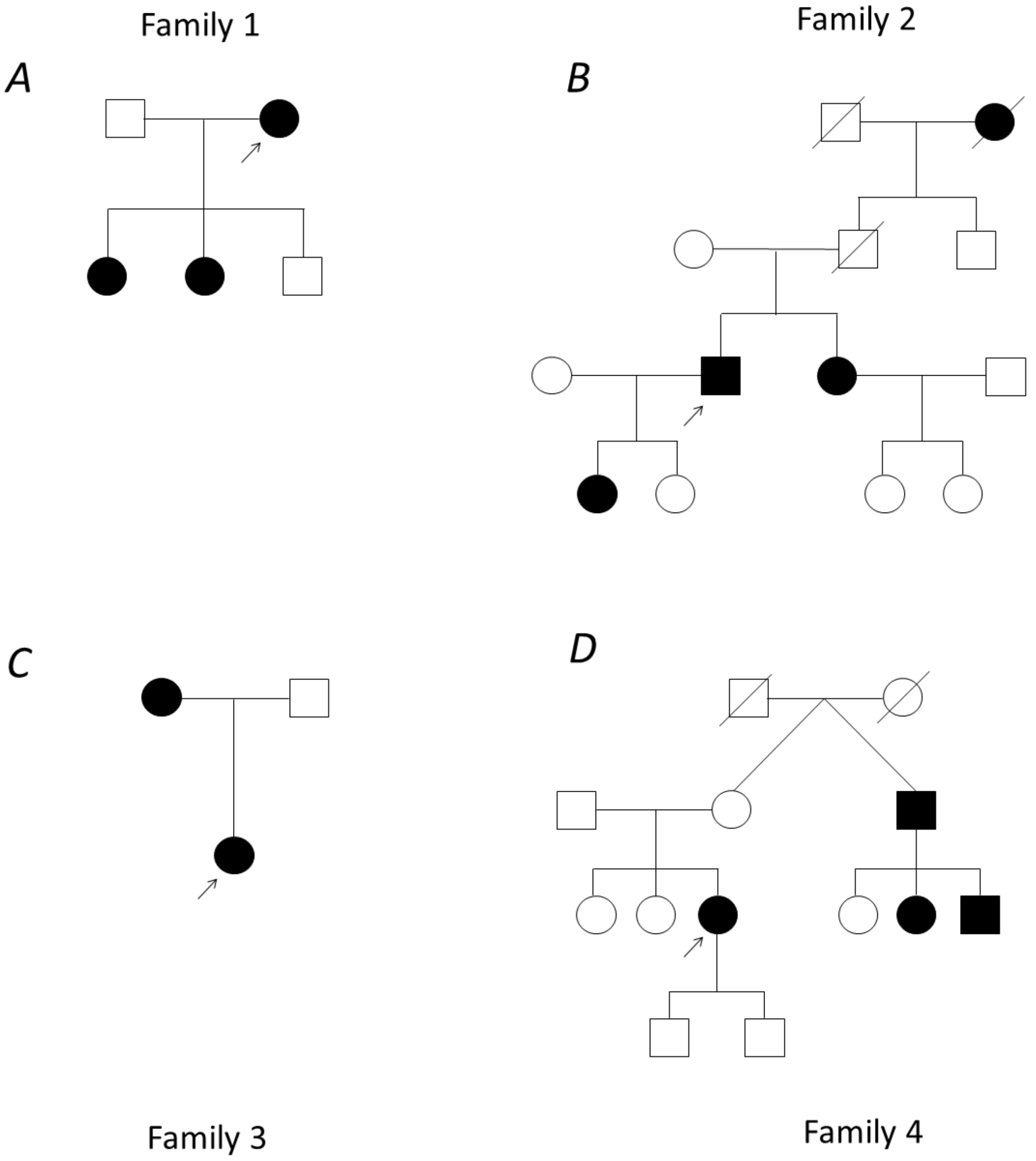

Supplementary Figure 1. Pedigrees for Families in whom more than 1 affected individual was examined (Families 1 to 4). Filled symbols denote affected individuals. *A*, Family 1: proband and her two daughters were examined; the proband underwent genetic testing. *B*, Family 2: proband and his sister were examined (and both underwent genetic testing); the proband reported that his paternal grandmother had some vision problems, becoming blind in her old age; no further details were known. The proband's elder daughter was also reported to have a similar macular appearance in one eye. *C*, Family 3: proband and her mother were examined; both underwent genetic testing. *D*, Family 4: proband and her affected maternal male cousin were

examined in our service (the proband’s affected maternal uncle and affected maternal female cousin were diagnosed elsewhere); only the proband underwent genetic testing. Results of examination and genetic testing are given in the main text and in Supplementary Table 1. No other individuals were examined or underwent genetic testing.
