# Supplementary material for: Clinical and Genetic Findings in CTNNA1-Associated Macular Pattern Dystrophy
Source: Ophthalmology. 2021 Jun;128(6):952–5. doi: 10.1016/j.ophtha.2020.10.032 (PMC8162661; doi:10.1016/j.ophtha.2020.10.032)
Supplement: Figure S2 [file mmc2.pdf]

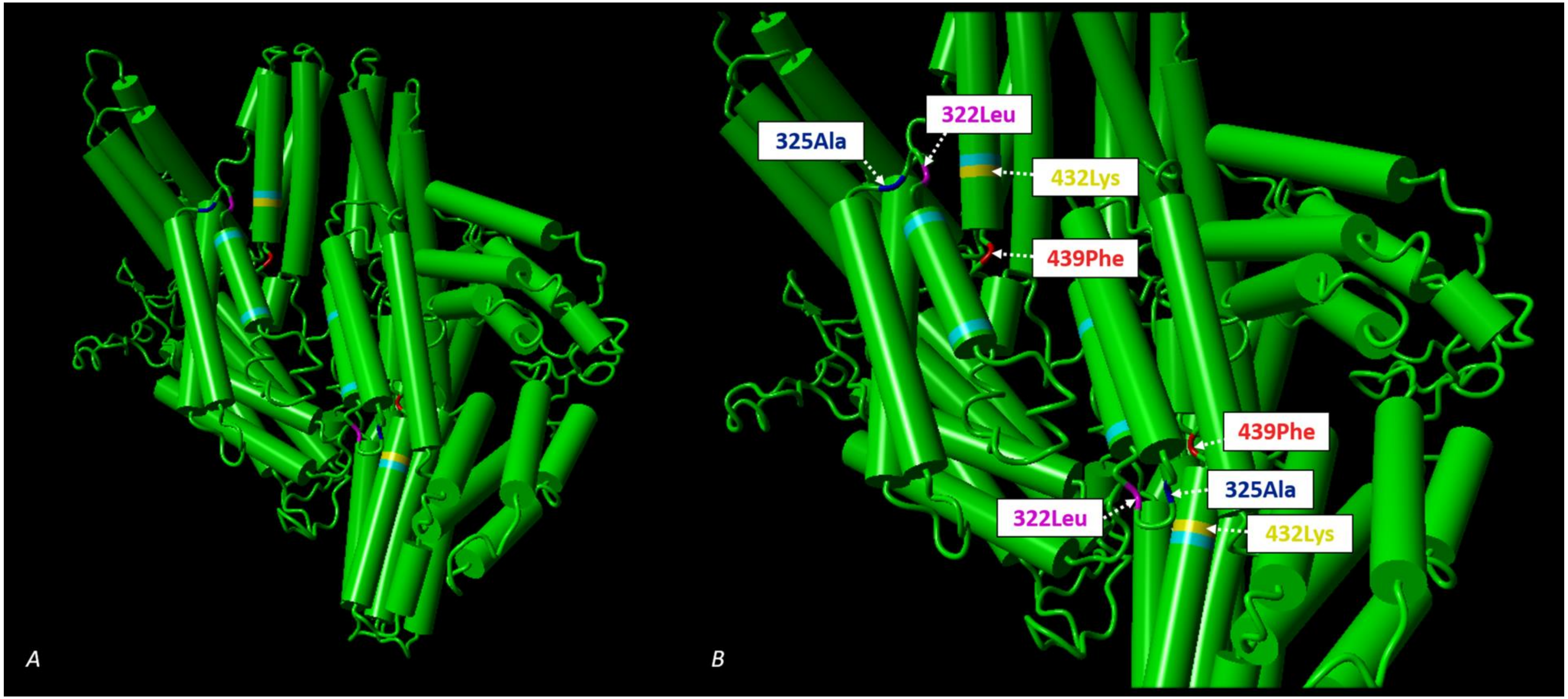

Supplementary Figure 2. Protein structure with positions of missense changes. *A*, Three-dimensional predicted protein structure with positions of previously reported changes[1] (shown in blue) as well as those reported in the current study. Three-dimensional structures were produced using online software (Protein Database 4IGG[2] and YASARA[3]). *B*, Magnified version of the region containing the changes, with amino acid changes from the current study labelled (positions 322, 325, 432 and 439; those identified in the previous study were at positions 307, 318 and 431). The c.965C>T and c.1316C>T changes result in substitution of the polar amino acid serine by leucine and phenylalanine respectively (both have hydrophobic side chains). The c. 1294G>A is predicted to change glutamate (negatively charged) to lysine (positively charged). The c.973A>G variant is predicted to change the polar amino acid

threonine to alanine (nonpolar, aliphatic). All four changes were predicted to be “probably damaging” by PolyPhen-2 and disease causing by MutationTaster. The latter predicted the variants to be evolutionarily distant and deleterious. Modelling depicts the variants on the side of the protein that interacts to form a dimer, suggesting dimerization might be compromised. Saksens et al.[1] noted that the disease-associated variants mapped in, or near to, the vinculin binding region; we speculate that the missense changes in our cohort might impact on vinculin binding as well, and lead to compromise of retinal pigment epithelium integrity.

References

1. Saksens N, Krebs M, Schoenmaker-Koller F, Hicks W, Yu M, Shi L, et al. Mutations in *CTNNA1* cause butterfly-shaped pigment dystrophy and perturbed retinal pigment epithelium integrity. *Nat Genet.* 2016;48(2):144-151.
2. Rangarajan E, Izard T. Dimer asymmetry defines alpha-catenin interactions. *Nat Struct Mol Biol*. 2013;20(2):188-193.
3. Krieger E, Vriend G. YASARA View—molecular graphics for all devices—from smartphones to workstations. *Bioinformatics*. 2014;30(20):2981–2982.
