# Supplementary material for: Clinical and Genetic Findings in CTNNA1-Associated Macular Pattern Dystrophy
Source: Ophthalmology. 2021 Jun;128(6):952–5. doi: 10.1016/j.ophtha.2020.10.032 (PMC8162661; doi:10.1016/j.ophtha.2020.10.032)
Supplement: Table S1 [file mmc3.pdf]

Family	*CTNNA1* variant	Individual	Age	Sex	Visual acuity	Fundus appearance	NIR-R	AF	OCT	ERG/EOG
1	c.965C>T p.(Ser322Leu)	Proband	43	F	Right 20/20 Left 20/17	Macular pigmentary clumping, peripheral reticular changes	Hyper-reflective areas	Linear areas of altered AF	Hyper-reflective subretinal deposit	ERG, PERG normal. EOG LP:DT ratio subnormal(150%).
		Daughter	4	F	Right 20/40 Left 20/40	Central foveal deposit	Foveal intense hyper-reflective lesion	Subtly irregular foveal AF	Hyper-reflective subretinal deposit	ERG, PERG normal (skin electrodes, undilated).
		Daughter	19	F	Right 20/20 Left 20/20	Subtle central altered foveal reflex	Foveal hyper-reflective lesion	No obvious abnormality	Subretinal deposits	ERG, PERG normal. EOG LP:DT ratio borderline (right 170%, left 165%).
2	c.1316C>T p.(Ser439Phe)	Proband	43	M	Right 20/17 Left 20/17	Linear areas of hyper and hypo-pigmentation radiating from foveal centre	Linear areas of hyper-reflectance	Linear areas of hyperAF	Disturbance at level of outer retina and RPE	ERG, PERG normal. EOG LP:DT ratio subnormal (right 135%, left 145%).
		Sister	38	F	Right 20/20 Left 20/20	Subtle pigmentary changes; peripheral areas of atrophy	NA	NA	NA	ERG, PERG normal. EOG LP:DT ratio subnormal (145%).
3	c.965C>T p.(Ser322Leu)	Proband	27	F	Right 20/20 Left 20/20	Pigmentary clumping	Hyper-reflectance in a wing-shaped pattern	Patches of increased AF	Hyper-reflective subretinal deposit	ERG, PERG normal.
		Mother	63	F	NA (asymptomatic)	Subtle pigmentary change and extramacular drusen	NA	Mild, variable hyper and hypo-AF	NA	NA
4	c.1316C>T p.(Ser439Phe)	Proband	32	F	Right 20/30 Left 20/17	Linear areas of pigmentary change	Linear areas of hyper-reflectance	Linear areas of hyperAF	Subretinal hyper-reflective deposit	ERG, PERG normal. EOG LP:DT ratio subnormal (right 125%, left 135%).
		Maternal cousin	34	M	Right 20/30 Left 20/20	Linear areas of pigmentary change	Linear areas of hyper-reflectance	Linear areas of hyperAF	Subretinal hyper-reflective deposit	ERG, PERG normal.
5	c.1294G>A p.(Glu432Lys)	Proband	31	F	Right 20/20 Left 20/20	Linear areas of pigmentary change	Linear areas of hyper-reflectance	Subtle patch of hyperAF	Subretinal hyper-reflective deposit	ERG, PERG normal.
6	c.973A>G p.(Thr325Ala)	Proband	62	M	Right 20/200 Left 20/30	Central atrophy in right eye and pigment mottling in left eye (later becoming atrophy)	Foveal hyper-reflectance and surrounding linear areas of hyper-reflectance	Foveal hypoAF in area of atrophy with surrounding linear or reticular areas of hyperAF	Outer retinal atrophy	ERG within normal limits (skin electrodes).* EOG normal (right 260%, left 200%).

Supplementary Table 1. Summary of genotypes and clinical features of the 11 patients. Families 1-5 were seen at Moorfields Eye Hospital; the proband from Family 6 was reviewed at the Manchester Centre for Genomic Medicine. The *CTNNA1* variants shown were identified by whole genome sequencing in Family 1 (proband, after specific testing of *PRPH2* found no causative variants), Family 2 (proband and sister, after specific testing of *PRPH2* and *BEST1* in proband was negative) and Family 4 (proband). In Family 3, samples were sent from the proband and her mother for screening of macular dystrophy genes (Molecular Vision Laboratory, Hillsboro, Oregon, Stargardt/Macular Dystrophy Panel, including *ABCA4*, *BEST1*, *CDH3*, *CERKL*, *DRAM2*, *EFEMP1*, *ELOVL4*, *IMPG1*, *IMPG2*, *PROM1*, *PRPH2*, *RP1L1*, *TIMP3*, *TTLL5*); no variants were found in these genes, but both patients were found to be heterozygous for the *CTNNA1* variant. In the proband of Family 5, screening of 577 genes associated with retinal dystrophies (Molecular Vision Laboratory, Hillsboro, Oregon) revealed heterozygosity for the above *CTNNA1* variant. In the proband of Family 6, screening of 176 retinal dystrophy genes (Manchester Centre for Genomic Medicine, retinal dystrophy panel) was negative; subsequent whole exome sequencing revealed the above heterozygous change in *CTNNA1*. No other variants were found in any other genes in any of the patients that could explain the phenotype. No other individuals were tested. Ages and visual acuities are at presentation. Electrophysiological testing was performed in most cases according to international standards. The EOG light peak to dark trough (LP:DT) ratios were measured, and compared with those in an unaffected control group (Mean - 2 standard deviations = 165%; reference interval 180% to 435%; n=30). NIR-R, near infrared reflectance; AF, autofluorescence (blue or green autofluorescence); OCT, spectral domain optical coherence tomography; ERG, electroretinogram (full-field); PERG, pattern electroretinogram; EOG, electro-oculogram; RPE, retinal pigment epithelium.

*For the Family 6 proband, full-field ERGs were recorded with skin electrodes and appeared within normal limits (with the possible exception of the dark-adapted strong flash ERG b:a ratio, considered equivocal).
